# Open access dataset of task-free hemodynamic activity in 4-month-old infants during sleep using fNIRS

**DOI:** 10.1038/s41597-022-01210-y

**Published:** 2022-03-25

**Authors:** Borja Blanco, Monika Molnar, Manuel Carreiras, César Caballero-Gaudes

**Affiliations:** 1 Basque Center on Cognition, Brain and Language (BCBL), Donostia - San Sebastián, Spain; 2 Department of Psychology, University of Cambridge, Cambridge, UK; 3 Department of Speech-Language Pathology, Faculty of Medicine, University of Toronto, Toronto, Canada

**Keywords:** Psychology, Psychology

## Abstract

Spontaneous, task-free, hemodynamic activity of the brain provides useful information about its functional organization, as it can describe how different brain regions communicate to each other. Neuroimaging studies measuring the spontaneous activity of the brain are conducted while the participants are not engaged in a particular task or receiving any external stimulation. This approach is particularly useful in developmental populations as brain activity can be measured without the need for infant compliance and the risks of data contamination due to motion artifacts. In this project we sought to i) characterize the intrinsic functional organization of the brain in 4-month-old infants and ii) investigate whether bilingualism, as a specific environmental factor, could lead to adaptations on functional brain network development at this early age. Measures of spontaneous hemodynamic activity were acquired in 4-month-old infants (n = 104) during natural sleep using functional near-infrared spectroscopy (fNIRS). Emphasis was placed on acquiring high-quality data that could lead to reproducible results and serve as a valuable resource for researchers investigating the developing functional connectome.

## Abstract (summary)

**Table:** 

Measurement(s)	spontaneous hemodynamic brain activity
Technology Type(s)	functional near infrared spectroscopy

## Background

Resting-state, or task-free, functional connectivity (FC) provides information about the intrinsic functional organization of the human brain. By measuring spontaneous fluctuations in cerebral hemodynamic activity FC can be used to identify temporally coherent activity patterns between brain regions, which support various functionally relevant sensory and cognitive processes (i.e., functional brain networks)^1,2^. FC can be used to examine differences in the maturational course of typical and atypical functional brain development as its development has been shown to be modulated by different pre- and postnatal factors^3–6^. This imaging approach is particularly appropriate for developmental populations. It does not require subject compliance with the task, as stimuli are not presented, and it can be recorded during natural sleep.

Task-free FC has been mostly measured by means of functional magnetic resonance imaging (fMRI), but this neuroimaging technique presents some limitations for testing developmental populations, such as scanner noise and the need of a rigid head stabilization. Functional near-infrared spectroscopy (fNIRS) is a non-invasive, noiseless, neuroimaging technique that measures the attenuation of NIR light as it travels through brain tissues to determine the relative concentration changes of oxyhaemoglobin (HbO) and deoxyhaemoglobin (HbR) within the sampled region. Such changes reflect the vascular response associated to neural activity and therefore represent a proxy measurement of functional brain activation^7^. FC studies in infants using fNIRS have demonstrated the ability of this technology to provide insights into the functional organization of the infant brain across development^8,9^. However, some caveats are inherent to any study using neuroimaging techniques on developmental populations, such as the presence of motion artifacts, short recording durations and small sample size.

Our project entitled ‘Group-level cortical functional connectivity patterns using fNIRS: assessing the effect of bilingualism in young infants’^10^ aimed to i) accurately describe group-level large-scale functional connectivity patterns in 4-month-old infants using high-quality fNIRS data collected in a large sample of participants, and ii) investigate if an early and continued bilingual exposure during the first months of life can lead to specific adaptations in functional brain network development that are observable under resting-state or ‘task-free’ conditions. This is the earliest age in development when differences between monolinguals and bilinguals have been observed. Concretely, behavioural studies showed differences in how infants from each language background allocate attention to language^11,12^, and neuroimaging works demonstrated dissimilar brain activation patterns during language related tasks^13,14^.

We used fNIRS to record oxy-haemoglobin (HbO) and deoxy-haemoglobin (HbR) spontaneous fluctuations across the cortex and investigate functional connectivity in 4-month-old infants from three different language backgrounds: Spanish monolingual, Basque monolingual, and Spanish-Basque bilingual infants. Participants were tested during natural sleep to maximize data quality and minimize attrition rate. Our sample consists of 104 participants with high-quality fNIRS data and long recordings of at least 9 min duration per participant.

## Methods

### Procedure

**Fig. 1:**
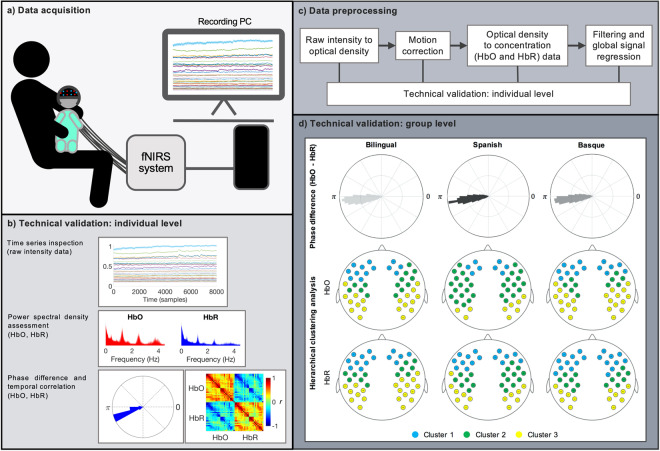
(**a)** Illustration of the fNIRS data acquisition process for the task-free experimental paradigm. (**b)** Data quality assessment indicators computed on all individual subjects. (**c)** Outline of the main steps of the fNIRS data preprocessing pipeline. (**d)** Technical validation performed at the group level in each of the three experimental groups.

The described dataset includes fNIRS data recorded in 104 healthy, full-term, 4-month-old infants. Spontaneous, task-free, hemodynamic activity of the brain was measured while infants slept leaning on their parents’ lap (Fig. 1a). Recordings were performed in a sound attenuated room where the only source of illumination was the screen of the recording computer attenuated to low brightness levels. Once the fNIRS cap was correctly positioned parents were asked to help their infants fall asleep. Recordings started when clear signs of sleep were noticeable by the parents and the experimenters (e.g., eyes closed, lack of movements). Parents were instructed to remain silent and to minimize movements over the duration of the recording to avoid involuntary cap or optode displacement. fNIRS measurements with a duration between 9 and 25 min were acquired.

Five infants were excluded for data analysis in our previously published project^10^ due to insufficient data quality (see Technical Validation, Fig. 1b), but these data are available in the repository for data quality evaluation purposes. The final sample included 99 participants belonging to three different language backgrounds: 36 Basque-Spanish bilingual infants (21 girls; mean age = 125 ± 4 days), 30 Spanish monolingual infants (13 girls; mean age = 123 ± 3 days) and 33 Basque monolingual infants (17 girls; mean age = 122 ± 4 days). Caregivers filled a questionnaire assessing infants’ language background. The questionnaire measured infants’ percentage of exposure to Spanish and Basque during the first months of life considering different contexts (e.g., weekday vs. weekend). The monolingual groups included participants that were exposed to a single language (Spanish or Basque), and those that presented less than 10% exposure to a second language. The bilingual group was formed by infants exposed to Spanish and Basque languages from birth, with the minimum percentage of exposure to the second language being higher than 10%. Individual information on gender, age, and percentage of exposure to Spanish and Basque is available in the repository (*BCBL_RS4_participant-info.csv*).

Infants wore a fNIRS headgear (NIRx NIRScout Medical Technologies, CA, USA) consisting of 16 light emitters and 24 detectors, containing a total of 52 channels for each haemoglobin oxygenation state, i.e., HbO and HbR. The system consists of two continuous wavelengths of source light at 760 and 850 nm with a sampling frequency of 8.93 Hz. The optode layout (i.e., sources and detectors) was positioned on a stretchy fabric cap (Easycap GmbH, Germany) according to the international 10–20 system using nasion, inion and preauricular points as external head landmark (see optode setup in online repository, *BCBL_RS4_optode-setup_reordered.pdf*). This configuration yielded source-detector separation distances ranging from approximately 20 mm to 45 mm, which covered frontal, temporal, parietal, and occipital regions of both hemispheres^10^. Caps of two different sizes, 40 and 42 cm, were employed to adapt to individual head size and shape. This approach ensured a consistent optode positioning across infants so that channels corresponded to analogous anatomical locations. During data collection for this study, an external 3D coordinate tracking system was not available. Therefore, 3D coordinates representing optode positions on each individual could not be included. Instead, the standard 10–20 positions of sources and detectors and the distance between them are provided (i.e., 2D coordinates). This information can be used to spatially register the fNIRS layout to age/size matched head models.

### Data preprocessing

The dataset has been uploaded in raw format and can be used as such with the user’s preferred data preprocessing pipeline. As reference, the main steps of the data preprocessing pipeline employed in our previously published study are outlined below (Fig. 1c). Preprocessed data, including data at different steps of the preprocessing pipeline, is also available in the online repository (*.mat* format).

Data preprocessing was performed in Matlab (R2012b and R2014b, MathWorks, MA, USA) using in-house scripts and third-party functions^15^. First, raw light intensity data were converted to optical density changes by computing the negative logarithm of the ratio between the detected light intensity and a reference baseline value (i.e., the mean signal). Noisy periods at the beginning or/and at the end of the recordings were visually identified and removed. Since data acquisition was performed during natural sleep, individual fNIRS recordings displayed high data quality. Some datasets showed sparse motion artefacts, which in fNIRS data are characterized by abrupt amplitude signal changes and/or artefactual signal drifts. A wavelet-based denoising method was applied to reduce motion artefacts^16^. After this step, optical density data was converted into HbO and HbR concentration changes by means of the modified Beer Lambert Law with differential pathlength factors of 5.3 and 4.2^17^. All datasets were manually cropped to 5000 samples (~560 sec), so that all infants contributed equally to subsequent steps of the analysis. A nuisance regression model including temporal filtering and global signal regression was applied to attenuate fNIRS signal contamination due to systemic fluctuations of global origin (e.g., respiration and cardiac pulse)^18^. Specifically, the regression model included Fourier terms for frequencies above 0.09 Hz to account for high-frequency physiological components. Slow frequency fluctuations and signal drifts were also modelled by 4th order Legendre polynomials (i.e., frequencies between 0 and 0.004 Hz approximately). Finally, the regression model also included the average fNIRS signal, which is assumed to largely represent global systemic hemodynamic changes^18,19^. As HbO and HbR are differently affected by global systemic processes, data of each haemoglobin chromophore were filtered independently by including either the average HbO or HbR signal in the regression model.

### Ethics

This study received approval from the local ethical committee at the Basque Center on Cognition, Brain and Language (Donostia - San Sebastián, Spain). Prior to participation, parents were informed about the aim of the study, the experimental procedures, and their right to withdraw from the study at any moment without providing a reason and with no negative consequences. They were also informed that non-identifiable data could be shared following open data recommendations. Written informed consent was obtained from all the parents prior to data acquisition.

## Data Records

Data are publicly available and can be accessed from the Open Science Framework (OSF) archive via 10.17605/OSF.IO/7FZKM (see^20^). Datasets have been uploaded in separated folders in five different formats (Raw files in NIRx format, Brain Imaging Data Structure - BIDS*, .snirf*, *.nirs* and* .mat*, see Table 1). Each of these folders contains the individual files corresponding to data from every participant. Anonymized participant IDs are consistent across data formats and with the participant information table and data quality assessment figures available at the repository. A description of the specific data structure for each data format is available in the repository (*BCBL_RS4_osf-data-format.rtf*).

**Table 1:** Summary of datasets.

Folder name	Data format	Description	Participants and paradigm
**raw**	NIRx	Raw data in Nirx format (.wl1,.wl2), and configuration information	4-month-old infants n = 104 38 Spanish-Basque bilinguals 34 Spanish monolinguals 32 Basque monolinguals Spontaneous hemodynamic activity measured during natural sleep. Recording duration ( > 9 min.)
**snirf**	*.snirf*	Raw data and fNIRS probe setup information	
**BIDS**	BIDS	Raw data and fNIRS probe setup information	
**nirs**	*.nirs*	Raw data and SD data structure	
**mat**	*.mat*	Raw data, preprocessed data, and SD data structure	

SD structure contains information of the fNIRS optode configuration: wavelengths, source-detector positions, and source-detector-channel combinations.

## Technical Validation

An individual data quality assessment routine was implemented as an initial technical validation before data preprocessing (Fig. 1b). Data quality indicators were selected based on previous guidelines on best practices for fNIRS data analysis^19^.

First, individual channel time series were inspected to detect motion-induced artifacts and signal drifts. This step was performed after each step of the preprocessing pipeline: i) raw intensity data, ii) optical density data uncorrected, iii) optical density data corrected, iv) HbO and HbR data, v) HbO and HbR data filtered, vi) HbO and HbR data after global signal regression (see data quality assessment in online repository, *BCBL_RS4_data-quality-assessment.pdf*).

Second, the presence of physiological components such as respiration and cardiac pulsation in the power spectral density of HbO and HbR prior to temporal filtering was evaluated. The presence of cardiac pulse in the power spectral density of HbO and HbR time series is considered one of the main indicators of good optode-scalp coupling^19,21^. These components show specific frequency patterns in infants, which are usually faster than those observed in adults. For example, cardiac pulse in infants is present at around 2 Hz, and a component related to respiration is usually observed at around 0.6 Hz.

Third, the phase difference and temporal correlation between HbO and HbR time series was characterized. These two parameters describe the intrinsic relationship between HbO and HbR hemodynamic time series, with an antiphase state and a strong negative correlation expected in high quality data. This relationship has been repeatedly observed in task-based and resting-state fNIRS studies^22–24^, and some works have employed these indicators on algorithms for noise detection or noise reduction methods^21,25^.

Finally, a group-level data quality assessment step was also included, where the results of two previous fNIRS RSFC infant studies were replicated. First, following the work by Watanabe *et al*. (2017)^24^, the expected antiphase state between HbO and HbR signals was demonstrated in each of our three experimental groups (Fig. 1d). Second, the work by Homae *et al*., (2010)^8^ was replicated by spatially grouping fNIRS channels based on the degree of similarity between their time series, measured as pairwise temporal correlation. In the analysis computed for HbO and HbR, and for each experimental group, similar spatial clusters as in the original study were obtained in bilateral frontal, temporal, and parietal regions (Fig. 1d). Quality assessment figures for each participant are available in the online repository^20^. Group-level replication analyses and results can be found in Blanco *et al*., (2021)^10^.

## Data Availability

Scripts to load the datasets into Matlab and run the described fNIRS data quality assessment routine are available at https://github.com/borjablanco/RS_4months. The rest of the Matlab functions used to analyse this dataset come from fNIRS data analysis package Homer2 (https://homer-fnirs.org/), and its more recent version Homer3 that can be found in (http://openfnirs.org/software/homer/). Interested readers are also directed to the *snirf* repository (https://github.com/fNIRS/snirf), which provides information for working with fNIRS data structured in this format. Information on how to convert a directory of fNIRS files to a correctly formatted BIDS dataset can be found in (https://github.com/rob-luke/fnirs-apps-sourcedata2bids).
